# Self-Reported Body Fat Change in HIV-Infected Men Is a Marker of Decline in Physical Health-Related Quality of Life with Aging, Independent of Co-Morbidity

**DOI:** 10.1371/journal.pone.0114166

**Published:** 2014-12-01

**Authors:** Kristine M. Erlandson, Sandra M. Reynolds, Christopher Cox, Frank J. Palella, Mallory D. Witt, Lawrence A. Kingsley, Todd T. Brown, Michael Plankey

**Affiliations:** 1 Department of Medicine, Divisions of Infectious Diseases & Geriatric Medicine, University of Colorado, Aurora, Colorado, United States of America; 2 Department of Epidemiology, Bloomberg School of Public Health, Johns Hopkins University, Baltimore, Maryland, United States of America; 3 Department of Medicine, Division of Infectious Diseases, Northwestern University, Chicago, Illinois, United States of America; 4 Department of Medicine, Division of HIV Medicine, Los Angeles Biomedical Research Institute at Harbor–University of California Los Angeles (UCLA), Torrance, California, United States of America; 5 Department of Infectious Diseases and Microbiology, University of Pittsburgh, Pittsburgh, Pennsylvania, United States of America; 6 Department of Medicine, Division of Endocrinology, Diabetes, and Metabolism, Johns Hopkins School of Medicine, Baltimore, Maryland, United States of America; 7 Department of Medicine, Division of Infectious Diseases, Georgetown University Medical Center, Washington, District of Columbia, United States of America; Tulane University School of Public Health, United States of America

## Abstract

**Objective:**

Self-perception of changes in body fat among HIV+ persons is associated with decreased health related quality of life in cross-sectional studies. The longitudinal impact of body fat changes on health related quality of life, while accounting for comorbidity and anatomic location or severity of body fat changes, is unknown.

**Design:**

This was a longitudinal analysis of HIV+ and HIV- Multicenter AIDS Cohort Study (MACS) participants who completed questionnaires assessing self-perceived body fat changes (baseline visit) and a health related quality of life (Short Form-36) at baseline and then ≥5 years later.

**Methods:**

Relationships between body fat changes and change in Short Form-36 Physical and Mental Component Summary scores were investigated using mixed-model regression.

**Results:**

We studied 270 HIV+ and 247 HIV- men. At baseline, ≥50% of HIV+ men reported body fat changes; physical component but not mental component summary scores were lower among HIV+ men who reported moderate/severe leg or abdominal fat changes (p<0.05). At follow-up, physical component summary scores were significantly lower among men with face, leg, or abdominal fat changes compared to men without perceived fat changes (p<0.05). No significant changes were seen in mental component scores by fat change location or severity. In the final model, body fat changes at any site or severity were significant predictors of a decline in physical component summary score (p<0.05), independent of demographics or comorbidities. Mental component summary score was not associated with body fat changes, but higher mental component summary score was associated with increasing age and time.

**Conclusions:**

Negative self-perceived body fat changes were associated with decline in physical health related quality of life, independent of comorbidities, and may be a marker of an increased risk for physical function decline with aging.

## Introduction

Abnormalities in body fat distribution among HIV-infected (HIV+) persons, including central lipohypertrophy and peripheral subcutaneous lipoatrophy, were recognized soon after the introduction of highly active antiretroviral therapy (HAART) [1]. While initially these abnormalities were thought to represent a single entity that was referred to as lipodystrophy, most data suggest that lipohypertrophy and lipoatrophy are distinct entities with separate etiologies and risk factors, although coexistence in an individual patient is common [2]–[4]. Both of these disorders are also independently associated with adverse metabolic consequences including dyslipidemia, insulin resistance, metabolic syndrome, and with increased levels of systemic inflammation or immune activation, all of which may increase the risk of cardiovascular disease, diabetes, and mortality [5]–[9].

In addition to the metabolic and inflammatory consequences, body fat changes are stigmatizing, and may impact the way in which HIV+ persons perceive their overall health and self-esteem. Persons affected by lipoatrophy or lipohypertrophy were more than twice as likely to feel that their body fat changes were noticed by others compared to persons without body fat changes [10], [11]. These negative perceptions can impact antiretroviral adherence and quality of life, as shown in prior studies [10], [12]–[16]. In one study, two-thirds of HIV+ participants reported that they would be willing to trade a year of life not to have “lipodystrophy” [17]. Many of the studies exploring the impact of perception of body fat changes on health-related quality of life (HR-QoL) measures have been small; few have differentiated between the effects or severity of lipoatrophy or lipohypertrophy [14], [15], [18], [19], few have included HIV-uninfected (HIV-) controls [20], and none have adjusted for other comorbid illnesses common with aging that may also impact HR-QoL. Furthermore, only one published study was longitudinal, and was limited to 37 men with lipoatrophy, lipohypertrophy or both, and had only two years of follow-up [20].

Although the incidence of HIV-associated lipoatrophy has decreased with earlier initiation of more modern, less toxic ART [21], the impact of self-perceived body fat changes on the HR-QoL among persons aging with HIV is not well understood. The goal of the present study was to determine the association between the presence and severity of self-perceived body fat changes in the face, leg or abdomen with physical and mental HR-QoL among HIV+ persons receiving HAART compared to HIV- persons.

## Methods

### Study Population

The Multicenter AIDS Cohort Study (MACS) is an ongoing multicenter prospective cohort study of the natural and treated histories of HIV infection among homosexual and bisexual men in the United States who are followed on a semi-annual basis. The study design has been described previously [22], [23]. Each semi-annual MACS study visit includes a comprehensive physical examination, blood specimen collection for CD4 cell count and plasma HIV-1 RNA determination, and an interviewer-administered questionnaire which provides data on demographics, disease characteristics, and specific HIV antiretroviral medication use. Additionally the HR-QoL Medical Outcomes Study Short Form-36 Health Survey (SF-36), the most widely used and best validated instrument of HR-QoL [24], is self-administered at each study visit. The institutional review boards at each site approved study protocols and forms, with approval by the Johns Hopkins University for the Center for the Analysis and Management of the Data from the Multicenter AIDS Cohort Study (CAMACS), IRB No: H.34.99.07.12.A.Each participant provided written informed consent.

HIV+ and HIV- participants who attended study visit 31 (April-October 1999), were considered for study inclusion. At this visit, both HIV+ and HIV- men were asked about the self-perception of body fat changes in the face (loss), legs (loss), and abdomen (gain) in the previous 2 years (“In the past 2 years, have you noticed any changes in the distribution or in the amount of your body fat [either loss or gain]?”), and were asked to assess the severity of the body fat changes at each anatomic site (“If ‘yes’, which parts of your body were affected and how severely?”). At each subsequent semi-annual visit, men were then asked whether perceived fat changes at these sites had been noted in the previous 6 months and if so, to rate the current severity. Men were categorized into one of the following categories of self-perceived body fat changes based on their response to the visit 31 questionnaire: “None”, “Mild”, “Moderate/Severe”. The visit 31 response was carried forward to subsequent semi-annual visits unless the participant reported a change at which point their response was updated.

To ensure that HIV+ men had been HAART-exposed at the time of the assessment of self-perceived body fat changes, we used responses provided at a semi-annual assessment 5 (+/−1) years after HAART initiation with concurrently existing SF-36 data. This visit was defined as the baseline visit. HAART was defined based on the Department of Health and Human Services guidelines as use of three or more antiretroviral medications from at least two classes including: a protease inhibitor, a non-nucleoside reverse transcriptase inhibitor, a nucleoside reverse transcriptase inhibitor, an integrase inhibitor, or an entry inhibitor [25].

### Outcome Measures

**Figure 1:**
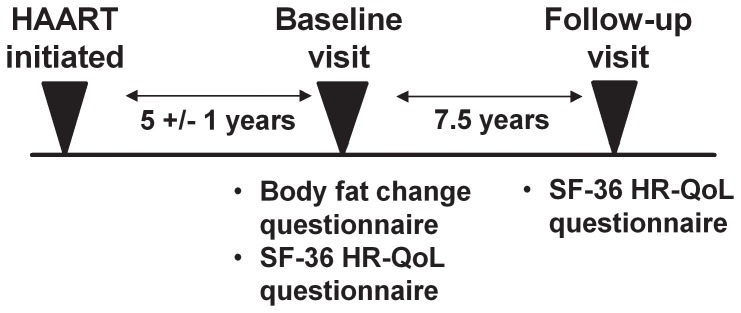
Study design indicating the relationship between the time highly active antiretroviral therapy (HAART) was initiated, the baseline visit where the initial Short Form (SF)-36 health related quality of life (HR-QoL) questionnaire was completed, and the follow-up visit when the second questionnaire was completed.

The HR-QoL data from the SF-36 can be summarized using a composite measure of physical and mental health. The physical health component score (PCS) summarizes 4 scales critical to physical HR-QoL: Physical Functioning, Role-Physical, Bodily Pain, and General Health. The mental health component score (MCS) summarizes 4 scales critical to mental HR-QoL: Vitality, Role-Emotional, Social Functioning, and Mental Health. Scores are normalized to a population mean of 50 with a standard deviation of 10 [26]. To examine the longitudinal change in HR-QoL, a follow-up visit was defined as the most recent visit at which the SF-36 was completed, and at least 5 years after the baseline visit (median duration 7.5 years), Figure 1.

### Independent Variables

HIV control was assessed by determining the proportion of post-HAART visits prior to the baseline or follow-up visits with plasma HIV-1 RNA <400 copies/ml. Hepatitis C virus (HCV) infection was defined as a detectable hepatitis C virus RNA level in serum; all others were considered uninfected with HCV. The comorbidity score was defined as the total number of the following comorbid conditions: 1) depression (Centers for Epidemiologic Studies Depression Scale score greater than 16 [27]), 2) hypertension (systolic blood pressure greater than 140 mmHg or diastolic pressure greater than 90 mmHg), 3) diabetes mellitus (fasting glucose greater than or equal to 126 mg/dL, or self-report of a clinical diagnosis with use of medication), 4) Dyslipidemia (either fasting total cholesterol greater than or equal to 200 mg/dL, low-density lipoprotein greater than or equal to 130 mg/dL, high-density lipoprotein less than 40 mg/dL, triglycerides more than or equal to 150 mg/dL, or use of lipid-lowering medications with self-report of a clinical diagnosis 5) kidney disease (estimated glomerular filtration rate less than 60 mL/min/1.73 m^2^ using the Modification of Diet in Renal Disease equation [28] or a urine protein-to-creatinine ratio greater than or equal to 200 mg protein/1 g creatinine), 6) liver disease (alanine transaminase or aspartate aminotransferase >150 IU/L), 7) cancer within 1 year (diagnosis at or within a year of the relevant visit [29]).

### Statistical Analysis

Mixed-model regression analysis of the HIV+ participants was performed to investigate the relationship between the severity of self-reported body fat changes (none versus mild versus moderate/severe) at the baseline visit and the PCS and MCS at the baseline and follow-up visits. The mixed model also included a time effect to assess the difference between the two time points, as well as the interaction between self-reported body fat changes and time. To examine specific differences among the 3 anatomic locations ×2 time points  = 6 groups, we made comparisons between pairs of adjusted means. Modeling was performed separately for the face, legs, and abdomen. All models were adjusted for age, race (Non-white versus White), education (college or more versus no college), HAART use at the time of the visit, co-morbidity score, and proportion of previous post-HAART visits with plasma HIV RNA (viral load) <400 copies/mL. For comparison we ran a similar analysis on the HIV- subgroup, adjusting for age, race, education, and comorbidities.

In a second analysis, we first used mixed models to determine the difference in PCS and MCS by HIV serostatus and evaluated whether the effect of HIV serostatus status differed over time. We then added the self-reported body fat change variable to the above model to determine whether the severity of body fat changes was related to physical and mental health-related quality of life and whether inclusion of this variable altered the effect of HIV serostatus. In a third model, we then added the co-morbidity score to the model to determine whether any of the effect related to HIV serostatus or body fat change was due to the burden of co-morbid conditions. All analyses were done using SAS (9.2).

## Results

**Figure 2:**
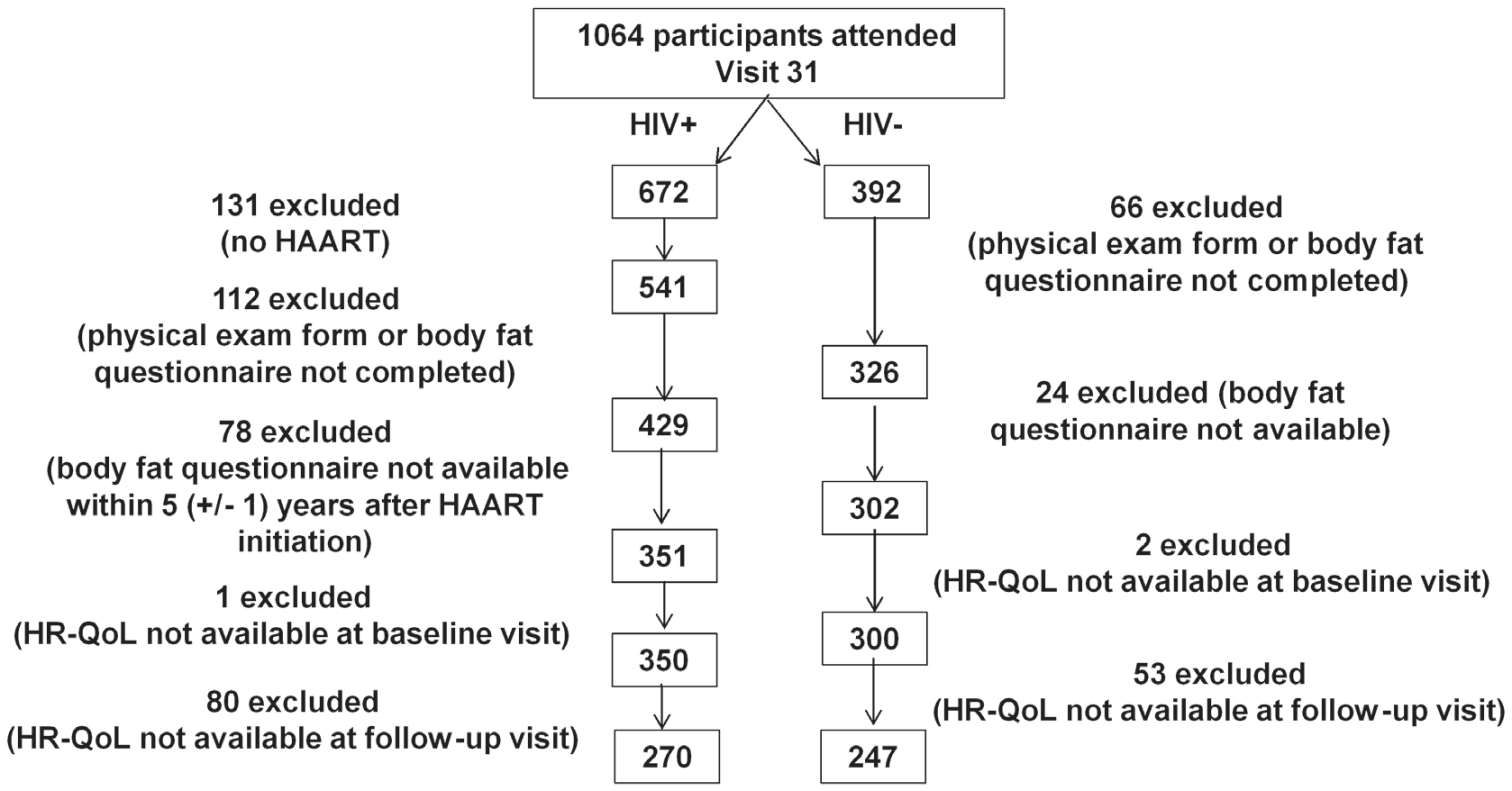
CONSORT diagram of study participant enrollment for HIV+ and HIV- participants. HAART, highly active antiretroviral therapy; HR-QoL, health related quality of life.

As shown in Figure 2, the analytic sample included 270 HIV+ and 247 HIV- men with non-missing data out of 1064 (672 HV+ and 392 HIV- men) who attended the baseline visit. The median time to the follow-up visit was 12.1 years after HAART initiation (IQR: 11.2, 12.7 years) and 7.5 years after the baseline visit (IQR: 6.9. 7.7).

Table 1 shows the participant characteristics at the baseline and follow-up visit. HIV+ men were slightly younger and had a lower body mass index than the HIV- men. Most of the men were white and most were college educated. The median number of comorbid conditions at the baseline visit was one for both the HIV+ and HIV- men, with the most prevalent conditions being dyslipidemia, depression, hypertension, and diabetes mellitus. The prevalence of diabetes mellitus, hypertension, dyslipidemia, and kidney disease increased over the follow-up interval for both HIV+ and HIV- men.

**Table 1:** Participant Characteristics at Baseline Visit (5 +/−1 years after Highly Active Antiretroviral Therapy [HAART] initiation) and Follow-up Visit (median 7.5 years later).

Characteristics	HIV+ (N = 270)		HIV- (N = 247)	
	Baseline	Follow-up	Baseline	Follow-up
Age, years	47 (43,52)		49 (45,54)	
Calendar year	2001 (2001, 2002)	2009 (2008, 2009)	2001 (2001, 2001)	2009 (2008, 2009)
White	224 (83%)		212 (86%)	
Body mass index, kg/m^2^	24.4 (22.6,26.9)		26.1 (24.0,29.4)	
College graduate	186 (69%)		185 (75%)	
Current smoker	48 (18%)		43 (17%)	
Nadir CD4 cell count, cells/uL	303 (187,461)			
Current CD4 cell count, cells/uL	544 (374,770)	557 (414,748)		
% visits with HIV-1 RNA <400 copies/mL	80 (40,100)	92 (60,100)		
Stavudine use	117 (43%)	11 (4%)		
Zidovudine use	96 (36%)	40 (15%)		
Protease inhibitor use	180 (67%)	151 (56%)		
Hepatitis C infection	17 (6%)	14 (5%)	3 (1%)	3 (1%)
Comorbidity score	1 (1,2)	1 (1,2)	1 (0,1)	1 (1,2)
Depression	61 (23%)	56 (21%)	41 (17%)	41 (17%)
Hypertension	44 (16%)	53 (20%)	38 (15%)	63 (26%)
Diabetes	27 (10%)	39 (14%)	9 (4%)	29 (12%)
Dyslipidemia	213 (79%)	219 (81%)	139 (56%)	190 (77%)
Kidney disease	5 (2%)	73 (27%)	0	10 (4%)
Liver disease	5 (2%)	4 (1%)	0	1 (0%)
Cancer within 1 year	3 (1%)	9 (3%)	2 (1%)	4 (2%)
Self-reported body fat change				
Lipoatrophy of face				
None	131 (49%)		229 (93%)	
Mild	76 (28%)		13 (5%)	
Moderate/severe	63 (23%)		3 (1%)	
Lipoatrophy of legs				
None	118 (44%)		226 (91%)	
Mild	75 (28%)		15 (6%)	
Moderate/severe	77 (29%)		4 (2%)	
Lipohypertrophy abdomen (%)				
None	101 (37%)		109 (44%)	
Mild	88 (33%)		85 (34%)	
Moderate/severe	81 (30%)		49 (20%)	

Values presented as number (%) or median (25^th^, 75^th^ percentile).

Co-morbidity score defined as the sum number of the following co-morbid conditions: 1) depression, 2) hypertension, 3) diabetes mellitus, 4) dyslipidemia, 5) kidney Disease, 6) liver disease, 7) cancer within 1 year.

Among the HIV+ men, the baseline median CD4 cell count was 544 cells/uL and HIV-1 RNA levels was undetectable at 80% of the visits since HAART initiation. At the follow-up visit, the median CD4 cell count was 557 cells/uL and HIV-1 RNA levels were undetectable at 92% of visits since HAART initiation. The use of stavudine, zidovudine, and protease inhibitors declined between the baseline and the follow-up visit.

Self-perceived fat changes at baseline were significantly different between HIV+ and HIV- men in the face (p<0.001), legs (p<0.001), and abdomen (p = 0.033). Over 50% of HIV+ men and less than 10% of HIV- men reported body fat changes in the face or legs. Sixty-three percent of HIV+ men and 54% of HIV- men reported self-perceived body fat changes in the abdomen (Table 1).

### Physical and Mental HR-QoL among the HIV+ Men: Effect of Time and Self-Perceived Body Fat Changes

At the baseline visit, PCS was not significantly different between men with or without facial fat changes (Table 2). In contrast, men who self-perceived moderate/severe body fat changes in the legs and abdomen had lower PCS compared to men with no self-perceived body fat changes at these sites. At the follow-up visit, men with any self-perceived body fat changes in the face, legs, or abdomen had significantly lower PCS than those without self-perceived body fat changes (p<0.05). Participants who did not initially report self-perceived body fat changes had stable PCS between the baseline and follow-up visits (Table 2). While there were trends towards significantly lower PCS between baseline and follow-up visits across all mild or moderate/severe self-perceived body fat change assessments, only the self-perception of moderate/severe body fat changes in the abdomen reached statistical significance (47.2 versus 44.9, p = 0.02). Over the same time interval, the HIV- men showed a significant decrease in PCS (baseline: 54.1 [95% CI: 48.9, 59.4] versus follow-up 52.0 [95% CI: 46.7, 57.3], p<0.001).

**Table 2:** Adjusted§ mean physical component summary (PCS) and mental component summary (MCS) scores on the Short Form-36 by self-reported severity of body fat changes at baseline and follow-up visit among HIV+ men.

Body Fat Change Location/Severity	PCS (Mean [95% CI])			MCS (Mean [95% CI])		
	Baseline Visit	Follow-up Visit	P value^†^	Baseline Visit	Follow-up Visit	P value^†^
*Face*						
None	49.9 (46.1, 53.7)	49.5 (45.7, 53.3)	0.61	48.3 (43.7, 52.8)	49.0 (44.5, 53.5)	0.43
Mild	47.6 (43.6, 51.7)	45.8 (41.7, 49.8)*	0.07	45.7 (40.9, 50.6)	46.4 (41.5, 51.2)	0.62
Moderate/Severe	47.4 (43.1, 51.7)	45.4 (41.1, 49.6)*	0.07	47.9 (42.8, 53.1)	48.9 (43.8, 54.0)	0.50
*Leg*						
None	50.1 (46.2, 53.9)	49.5 (45.7, 53.3)	0.48	47.6 (43.0, 52.2)	48.8 (44.2, 53.4)	0.23
Mild	48.0 (43.9, 52.0)	46.1 (42.1, 50.1)*	0.06	47.5 (42.7, 52.4)	47.3 (42.5, 52.1)	0.86
Moderate/Severe	47.1 (42.8, 51.3)*	45.7 (41.5, 49.8)*	0.17	47.4 (42.3, 52.5)	48.5 (43.4, 53.5)	0.42
*Abdomen*						
None	50.5 (46.6, 54.4)	50.3 (46.4, 54.2)	0.78	47.0 (42.3, 51.7)	47.5 (42.8, 52.1)	0.64
Mild	48.4 (44.5, 52.4)	47.2 (43.3, 51.2)*	0.19	48.9 (44.1, 53.6)	48.5 (43.8, 53.3)	0.77
Moderate/Severe	47.2 (43.1, 51.3)*	44.9 (40.8, 48.9)*	0.02	46.2 (41.3, 51.1)	48.7 (43.8, 53.5)	0.05

§ All models were adjusted for age, race (white vs other), education (college vs no college), HAART use at the time of the visit, co-morbidity score, and proportion of previous post-HAART visits with plasma HIV-1 RNA (viral load) <400 copies/ml.

95% CI: 95% Confidence Interval; † p value comparing baseline to follow-up; * p value <0.05 compared within visit to category “none”

MCS were similar by the severity of body fat changes at all three anatomic sites in the HIV+ men at both the baseline and follow-up visits (Table 2). Men who reported moderate/severe body fat changes in the abdomen tended to have higher MCS at the follow-up visit compared to baseline (46.2 versus 48.7, p = 0.053). Over the same time interval, the HIV- men showed a significant increase in MCS (baseline: 51.8 [95% CI: 45.0, 58.6] versus follow-up 54.0 [95% CI: 47.1, 60.8]; p = 0.004).

### Physical and Mental HR-QoL in HIV+ and HIV- Men: Effect of HIV Serostatus, Time, Self-Perceived Body Fat Changes and Comorbidity

The effect of HIV serostatus and time on PCS without consideration of the self-perceived body fat changes was first examined (Table 3; Model 1). Overall, PCS was 3.89 points (95% CI: −5.45, −2.33) lower in the HIV+ than the HIV- men. Increasing age and time (follow-up versus baseline visit) were associated lower with PCS, but there was no difference in the magnitude of the PCS decrease between baseline and follow-up visits by HIV serostatus. Inclusion of the severity of body fat change into the regression model decreased the effect estimates of HIV serostatus in the models of face or leg fat changes (47% and 48% decreases respectively), but had minimal effect on self-perceived abdominal body fat changes (7% decrease in the HIV serostatus effect estimate). PCS was significantly lower with increasing severity of self-perceived body fat changes at the all three sites (face, leg, and abdomen). The results for the HIV serostatus effect and the self-perceived body fat change severity effect were similar when the comorbidity score was added to the regression models (Table 3), although a higher comorbidity score was associated with lower PCS in all models.

**Table 3:** Change in Short Form-36 Physical Component Summary (PCS) and Mental Component Summary (MCS) Scores: Effect of HIV & Time, Self-Perceived Body Fat Changes, and Comorbidity.

	Model 1	Model 1+ Body Fat Change Severity			Model 1+ Body Fat Change Severity + Comorbidity		
	Estimate (95%CI)	Estimate (95% CI)			Estimate (95% CI)		
*Physical Component Score*		Face	Legs	Abdomen	Face	Legs	Abdomen
Age† (years)	−0.16 (−0.26,−0.07)**	−0.16 (−0.26,−0.07)***	−0.16 (−0.26,−0.07)***	−0.15 (−0.25,−0.05)**	−0.16 (−0.25,−0.06)**	−0.16 (−0.25,−0.06)**	−0.14 (−0.24,−0.05)**
HIV+ (vs HIV−)	−3.89 (−5.45,−2.33)***	−2.08 (−3.80,−0.36)*	−2.03 (−3.78,−0.29)*	−3.62 (−5.18,−2.06)***	−1.82 (−3.54,−0.11)*	−1.77 (−3.51,−0.03)*	−3.29 (−4.85,−1.72)***
Time (follow-up vs baseline)	−2.16 (−3.22,−1.11)***	−2.17 (−3.23,−1.11)***	−2.21 (−3.27,−1.15)***	−2.16 (−3.22,−1.09)***	−1.84 (−2.93,−0.74)**	−1.88 (−2.96,−0.79)***	−1.83 (−2.92,−0.73)**
Time x HIV+	0.83 (−0.63,2.29)	0.84 (−0.63,2.30)	0.88 (−0.58,2.34)	0.83 (−0.64,2.30)	0.76 (−0.71,2.23)	0.81 (−0.66,2.27)	0.75 (−0.72,2.22)
Mild vs no fat change		−3.60 (−5.55,−1.66)***	−3.26 (−5.21,−1.31)**	−1.91 (−3.47,−0.36)*	−3.45 (−5.38,−1.52)***	−3.04 (−4.98,−1.10)**	−1.76 (−3.31,−0.21)*
Mod/severe vs no fat change		−4.22 (−6.45,−1.99)***	−4.29 (−6.39,−2.18)***	−3.55 (−5.26,−1.85)***	−3.97 (−6.18,−1.75)***	−4.11 (−6.19,−2.02)***	−3.33 (−5.03,−1.63)***
Comorbidity score					−0.74 (−1.29,−0.19)**	−0.76 (−1.31,−0.21)**	−0.75 (−1.30,−0.20)**
*Mental Component Score*							
Age† (years)	0.24 (0.12,0.37)***	0.24 (0.12,0.36)***	0.25 (0.13,0.37)***	0.25 (0.12,0.37)***	0.27 (0.16,0.39)***	0.28 (0.16,0.40)***	0.28 (0.16,0.39)***
HIV+ (vs HIV−)	−0.68 (−2.62,1.27)	0.71 (−1.46,2.89)	0.17 (−2.03,2.38)	−0.48 (−2.45,1.50)	1.84 (−0.25,3.93)	1.30 (−0.81,3.41)	0.97 (−0.93,2.87)
Time (follow-up vs. baseline)	0.22 (−1.06,1.51)	0.23 (−1.07,1.52)	0.31 (−0.98,1.59)	0.26 (−1.03,1.56)	1.67 (0.39,2.94)*	1.75 (0.48,3.02)**	1.72 (0.44,3.00)**
Time x HIV+	−0.27 (−2.05,1.51)	−0.28 (−2.06,1.51)	−0.36 (−2.13,1.42)	−0.32 (−2.10,1.47)	−0.61 (−2.33,1.10)	−0.68 (−2.40,1.03)	−0.66 (−2.37,1.06)
Mild vs no fat change		−3.27 (−5.74,−0.79)**	−2.43 (−4.92,0.05)	−0.29 (−2.29,1.71)	−2.62 (−4.98,−0.25)*	−1.49 (−3.85,0.88)	0.39 (−1.51,2.30)
Mod/Severe vs no fat change		−2.63 (−5.47,0.21)	−1.32 (−3.99,1.36)	−1.18 (−3.37,1.01)	−1.54 (−4.25,1.18)	−0.54 (−3.09,2.01)	−0.21 (−2.30,1.88)
Comorbidity Score					−3.23 (−3.89,−2.58)***	−3.27 (−3.92,−2.62)***	−3.28 (−3.93,−2.62)***

95% CI: 95% confidence interval; Comorbidity score: the sum number of the following co-morbid conditions: 1) depression, 2) hypertension, 3) diabetes mellitus, 4) dyslipidemia, 5) kidney disease, 6) liver disease, 7) cancer within 1 year;

† age at baseline;

*p<0.05;

**p<0.01;

***p<0.001.

In the fully adjusted model, both higher baseline age and time were associated with higher MCS, suggesting an improvement in mental HR-QoL with increasing age (Table 3). There was no difference in MCS by HIV serostatus. Increasing comorbidity burden was strongly associated with lower MCS in all models (all p<0.001). Severity of body fat change was not associated with differences in MCS, with the exception that mild facial body fat changes were associated with significantly lower MCS compared to no facial fat changes (−2.62 [95%CI: −4.98,−0.25]; p<0.05).

## Discussion

HR-QoL is a multidimensional concept that focuses on the effects of health status on one's self-perceived HR-QoL. Although the long-term consequences of HIV-related body fat changes on metabolic and cardiovascular risk are medically well-appreciated, ours is the first analysis reporting upon of the impact of self-perceived body fat changes on the change in HR-QoL over a median of 7.5 years, adjusted for both HIV serostatus and comorbidity score. We have demonstrated that body fat changes of any severity or anatomic location were strongly associated with physical components of HR-QoL, independent of the impact of HIV serostatus and comorbid diseases. Although the impact of body fat changes on PCS increased with severity, participants that perceived only minor changes were not immune to these effects. A difference of 2–3 points in PCS score, which was seen in persons with minor self-perceived body fat changes, is similar to the decreases seen for each decade of advancing age across a normative population [30]. Furthermore, the PCS observed in our group with moderate to severe self-perceived body fat changes at follow-up was similar to the mean PCS in a normative population at least a decade older (65–74 years of age). Importantly, PSC have been consistently stronger predictors of mortality than MSC across multiple cohorts of HIV- participants [31], [32], thus our findings may have significant clinical implications in terms of mortality risk among persons with lipoatrophy or lipohypertrophy.

The greater decline in physical HR-QoL observed among HIV+ men with self-perceived body fat changes also suggests that these men are at an increased risk for decline in physical function with aging compared to HIV- men. This hypothesis is supported by findings from prior cross-sectional studies demonstrating an association between body fat changes and a frailty-type phenotype [33], and a separate longitudinal study that found lipoatrophy, lipohypertrophy, or both were predictive of a decline in grip strength [34]. The impact of body fat changes on physical function is likely multifactorial: lipoatrophy may serve as a marker for use of prior, more toxic ART associated with mitochondrial damage and impaired exercise tolerance [35], [36]. Lipoatrophy may be associated with a higher resting energy expenditure [37] and result in greater levels of fatigue. Furthermore, impaired skeletal muscle glucose uptake in lipoatrophy and lipohypertrophy may limit exercise duration and intensity [38]; heightened levels of inflammation that have been associated with body fat changes may directly impair skeletal muscle [39]; and the loss of lower extremity fat may be accompanied by a loss in lower extremity muscle mass [40].

In contrast to our expected findings, the mental health components of HR-QoL were preserved among HIV+ men with self-reported lipoatrophy or lipohypertrophy of any severity. Although many prior studies have reported an association between mental health and body fat changes, our lack of significant findings here may reflect the selectivity of the domains of mental health assessed in the different study questionnaires. Many prior studies explored specific domains of sexual function, body image, or depressive symptomatology that are not assessed by the more general mental health assessment of the SF-36 [15], [18], [19]. Of note, we also failed to observe differences in SF-36 by the location of self-perceived body fat changes, in contrast to prior studies that have shown a stronger association between mood changes and facial lipoatrophy than with abdominal or leg fat changes [18]. Significant improvements in SF-36 scores, Beck Depression Index Scores, body image, and self-esteem have been demonstrated with treatment of facial lipoatrophy among HIV+ persons [41], [42].

Not only did we fail to see a detrimental effect of self-perceived body fat changes on MSC, we observed significant overall improvement in the MSC scores with advancing age among both HIV+ and HIV- men, similar to reported observations across normative SF-36 values [30]. Prior studies have reported that older adults with HIV may have fractured social networks due to HIV/AIDS stigma or ageism [43], and that depression rates among HIV+ adults increase with advancing age [44], leading to our expectation of a decline in MCS scores with increasing age. We postulate that one possible explanation of our findings is that the stigma of lipoatrophy and lipohypertrophy is reduced as the body fat changes are attributed to aging rather than HIV infection. The loss of facial or leg fat and gain in central fat mirror age-associated changes in body fat albeit often appearing at earlier ages [45], [46]. Furthermore, the increase in MSC may represent greater resilience in our participants [47], [48], including resolved internalized homophobia [49], or that the potentially stigmatizing effects of lipoatrophy and lipohypertrophy are of little importance in the current ART era.

Our study has several limitations. First, the study population included only men. HIV+ women may have different or more pronounced manifestations of lipoatrophy and lipohypertrophy with differential effects on mental and physical HR-QoL [3], [14], [19]. Second, we used a measure of self-reported body fat changes rather than physician-diagnosed changes in body fat. Although self-reported body fat changes correlate well with physician-diagnosed changes, self-reported changes are consistently more common and more severe than provider-diagnosed changes and may be a more sensitive measure of subtle changes in body fat [2], [15]. The questionnaire that we used to assess self-perceived body fat changes was similar to questionnaires used in other studies that featured an inclusive of a range of responses to either an increase or decrease in body fat from none, mild, moderate, to severe. For this study, we evaluated only the changes in face, legs, or abdomen, and therefore may have missed effects of other self-perceived fat changes such as dorsocervical fat pads or arm lipoatrophy. Lastly, although the MACS men were diverse in terms of age, socioeconomic status, race/ethnicity and geographic region, they may not be nationally representative of HIV+ or HIV- men.

The main strength of our study is the longitudinal data collection: all exposures were ascertained consistently across the cohort over time for both HIV+ and HIV- men. Due to the size of our study population, we were able to adjust for age, HIV serostatus, and comorbidity score, ensuring that the analysis of HR-QoL changes were not due to these common confounders of HR-QoL. We determined the impact of body fat changes at three different anatomic sites, each of which could have different effects on perceptions of health [15], [18].

In summary, we found a strong association between self-perceived body fat changes and declines in physical but not mental HR-QoL, independent of the effects of chronologic age, calendar time, and comorbidity burden over 7.5 years. Among HIV+ men, self-perceived body fat changes may be a marker of increased risk for physical function decline with aging. Interventions to preserve physical function with aging could be informed by investigating the interrelationship of objective body fat changes, self-perception of body fat changes, and physical function.
